# Sputum IgE and Cytokines in Asthma: Relationship with Sputum Cellular Profile

**DOI:** 10.1371/journal.pone.0058388

**Published:** 2013-03-26

**Authors:** Maïté Manise, Gabriele Holtappels, Koen Van Crombruggen, Florence Schleich, Claus Bachert, Renaud Louis

**Affiliations:** 1 Department of Pneumology, GIGA I^3^Research Unit, Centre Hospitalier Universitaire du Sart-Tilman, Liege, Belgium; 2 Upper Airway Research Laboratory, ENT Department, Ghent University Hospital, Ghent, Belgium; Centro di Riferimento Oncologico, IRCCS National Cancer Institute, Italy

## Abstract

**Background:**

Local IgE production may play a role in asthma pathogenesis. The aim of the study was to assess sputum total IgE and cytokines in asthmatics according to sputum cellular phenotype.

**Methods:**

We studied 122 subjects including 22 non atopic healthy subjects, 41 eosinophilic (sputum eosinophils ≥3%), 16 neutrophilic (sputum neutrophils >76%) and 43 pauci-granulocytic asthmatics (sputum eosinophils <3% and sputum neutrophils ≤76%) recruited from the asthma clinic at CHU Liege.

Sputum supernatant total IgE (tIgE) was measured by ImmunoCAP and sputum supernatant cytokines (IL-4, IL-5, IL-6, IL-10, IL-13, IL-17, IFN-γ and TNF-α) were measured with the Luminex xMAP Technology by using commercially available Fluorokine MAP kits.

**Results:**

After concentrating sputum samples, total IgE was detectable in the majority of subjects. Sputum IgE was raised in asthmatics when compared to healthy subjects. Overall, asthmatics did not significantly differ from healthy subjects with respect to cytokine levels. The eosinophilic asthma phenotype, however, was characterised by raised sputum tIgE, IL-5 and IL-13 compared to healthy subjects (p<0.001, p<0.001 and p<0.05 respectively) and pauci-granulocytic asthma (p<0.01, p<0.001 and p<0.05 respectively) and raised IL-5 compared to neutrophilic asthma (p<0.01). When patients were classified according to sputum IgE levels, it appeared that IL-5, IL-6, IL-17 and TNF-α sputum supernatant levels were raised in the “IgE high” asthmatics (IgE ≥0.1 kU/l) when compared to “IgE low” asthmatics (IgE<0.1 kU/l).

**Conclusion:**

The eosinophilic asthma phenotype was associated with raised sputum IgE and a Th2 cytokine profile. Raised sputum IgE was associated with a heterogeneous cytokine overproduction.

## Introduction

It is now recognised that asthma actually comprises several inflammatory phenotypes and Simpson has proposed to break down asthma according to the granulocyte fraction contained in sputum cells [1]. Asthma is generally seen as an eosinophilic disease [2]. However, several studies showed that a fraction of asthmatic patients who exhibited the clinical symptoms of asthma and airway hyperresponsiveness do not have raised sputum eosinophils [3] and that this non-eosinophilic pattern of inflammation occurs across the all spectrum of severity [4]–[6]. A fraction of non-eosinophilic asthmatics actually exhibit raised airway neutrophilic inflammation. Those patients with non-eosinophilic asthma appear to be relatively resistant to corticosteroid therapy and are likely to recognize different underlying molecular mechanisms [7].

Local production of IgE might not be reflected by serum IgE or atopic status. Very recent data have shown that tIgE and specific IgE may be measurable in sputum from asthmatics irrespective of their atopic status even if their ability to prime local mast cells is still unclear [8]. However, it has also been demonstrated that local IgE in nasal polyp samples is functional [9] and is associated with comorbid asthma [10]. Monomeric IgE binding to its high affinity receptor FcεRI results in cell activation and survival independent of the presence of any allergen [11]; [12]. This makes of local IgE an important mediator in the mast cell activation pathway.

Recent studies demonstrated that total IgE in asthmatics is related to specific IgE against Staphylococcus aureus enterotoxins, which is found to be highly frequent in severe asthmatics independent of the atopic status [13]–[15]. Interestingly, spec IgE to Staphylococcus aureus enterotoxins is associated with lower FEV1, and higher intake of oral glucocorticosteroids and hospitalisation due to asthma exacerbations.

How local IgE production is related to the airway cellular inflammatory profile remains poorly studied. It is assumed that IgE production is tightly regulated by the balance between Th1 and Th2 cytokines, interleukin-4 and 13 being involved in the immunological switch towards IgE [16]. Il-5 is a cytokine recognized to be critical in promoting eosinophilic inflammation [17]. Beside classical Th2 profile there has been recent interest for the IL-17 pathway in asthma and in particular in severe neutrophilic asthma [18]. Whether IL-17 pathway and neutrophilic asthma are related to disease severity and local IgE synthesis has not been studied so far. Classically, IL-6 has been viewed as a pro-inflammatory cytokine. Recent advances have documented a series of IL-6 activities that are critical for resolving innate immunity and promoting acquired immune responses [19]. TNF-α is a potent pro-inflammatory cytokine that favours granulocytes recruitment and which as been associated with asthma pathogenesis [20].

The purpose of our study was to assess tIgE (sputum supernatant total IgE) , serum IgE and sputum cytokines in a large sample of asthmatics classified according to their sputum cellular phenotype. We also aimed to determine whether IgE and cytokines were related to disease severity or atopy.

## Materials and Methods

### Study design and subjects characteristics

Patient demographic, functional and treatment characteristics are given in table 1. In this study we enrolled 100 subjects consecutively recruited from our asthma clinic at CHU Liege (41 eosinophilic, 16 neutrophilic and 43 pauci-granulocytic asthmatics). All asthmatics were diagnosed on the basis of significant FEV1 reversibility (≥12% from baseline) to β2-agonists or bronchial hyperresponsiveness to methacholine (PC20 M<16 mg/ml). Atopy was defined as a positive skin prick test reaction (weal ≥3 mm compared with control) to common aeroallergens including house dust mites, cat and dog dander, grass, tree, pollen and moulds. Different groups of asthmatics were compared to 22 non atopic healthy subjects. The eosinophilic asthma phenotype was defined by a sputum eosinophil count ≥3% while sputum was considered to be neutrophilic when neutrophil count exceeded 76% (mean +1.7 SD of neutrophil count derived from a population of 113 healthy subjects with a mean age of 37 years). Contrary to eosinophils, the range of the upper limit of the 90% confidence interval for neutrophils can considerably vary according to the different labs (from 49% to 93%) [21]. Those who had less than 3% eosinophil count and less than 76% neutrophil count were considered as pauci-granulocytic. Those with eosinophil count >3% and neutrophil count >76% were considered as mixed granulocytic but discarded from further analysis because only 3 patients satisfied these criteria. A subgroup of our asthmatic population was considered as refractory asthmatics (N = 35). They were defined according to the ATS criteria and had been followed for at least 6 months in our department and received education about their disease before entering this study.

**Table 1:** Demographic, functional, airway inflammatory and treatment characteristics according to sputum cellular profile.

	Healthy subjects (N = 22)	Eosinophilic asthma (N = 41)	Neutrophilic asthma (N = 16)	Pauci-granulocytic asthma (N = 43)
Age (years)	42±13	54±11*	51±17	38±14 †††
Sex (m/f)	14/8	26/15	5/11	20/23
Tobacco status (ns/es/cs)	15/3/4	18/16/7	10/4/2	24/9/10
Pack-year	16±11 (N = 7)	19±21 (N = 19)	42±17 † (N = 6)	16±14 ‡ (N = 18)
BMI	25±6	27±4	26±6	26±5
Atopy	0	23 (56%)	7 (43%)	25 (58%)
FENO_50_ (ppb)	21 (6–48)	52 (9–222)*	20 (6–200)	16 (5–81) †††
FEV1 (%)	103±16	82±26**	70±25***	90±16 *
FVC (%)	108±13	95±22	83±20***	97±13
FEV1/FVC (%)	81±7	70±13**	67±15*	76±10
Reversibility (%)	–	16±19	9±12	10±15
PC20M (mg/ml)	> 16 mg/ml	0.81 (0–15)	0.46 (0.1–14)	0.56 (0.2–14)
ACQ	ND	1.6 (0–5.1)	2.1 (0–4.7)	1.7 (0–4.4)
Blood eosinophils (%)	1.7 (0.7–6)	5 (2–24)***	2 (0.2–5)††	2 (0.3–9) †††
Blood neutrophils (%)	53 (47–69)	52 (40–65)	63 (50–79)††	55 (42–72)
Sputum eosinophils (%)	0 (0–11)	14 (3–89)***	0.2 (0–2.7)†††	0.5 (0–2.6) †††
Sputum neutrophils (%)	35 (0–88)	37 (3–68)	91 (80–100)*** †††	47 (0–76) ‡‡‡
ICS	0	26 (63%)	11 (69%)	25 (58%)
ICS (eq becl/day)	0	2000 (400–2000)	2000 (500–4800)	1000 (400–3000)
LABA	0	21 (51%)	10 (62%)	18 (42%)
LTRA	0	8 (20%)	1 (6%)	9 (21%)
Theophylline	0	2 (4.9%)	3 (19%)	2 (4.6%)
Hospi/patient/year	0	0.24±0.43	0.25±0.44	0.19±0.39
Exacerbation/patient/year	0	1.09±1.93	0.81±1.64	0.9±2.07
Oral CS≥50% time	0	1 (2.4%)	2 (12.5%)	2 (4.6%)

Age, BMI, lung function, hospi/patient/year and exacerbation/patient/year are expressed as mean ± SD, PC20M as geometric mean and other parameters as median (range), becl = beclomethasone, * p<0.05, ** p<0.01, *** p<0.001 vs healthy subjects; † p<0.05, †† p<0.01, ††† p<0.001 vs eosinophilic; ‡ p<0.05, ‡‡ p<0.01, ‡‡‡ p<0.001 vs neutrophilic. ND = not defined.

The protocol had been approved by the local Ethics Committee (Hospital-Faculty ethics committee of Liege University) and every subject gave his written informed consent.

### Peripheral blood sampling, serum IgE and cell count measurement

Peripheral blood samples were collected in serum tubes with gel (Venosafe, TERUMO®, Belgium). Tubes were centrifuged at 800 *g* for 10 min at 4°C and sera were conserved into aliquots at −80°C until assay. The total and differential blood cell counts were obtained with an Advia 210 automatic counter (USA). Counting and cell typing were based on flow cytometry with bidimensionnal volume distribution, peroxydase concentration and lobularity of leukocytes as parameters. Serum total IgE, serum specific IgE against staphylococcus aureus and serum specific IgE against the most common aeroallergens were measured with the ImmunoCAP system with a detection limit of 2 kU/l, 0.1kU/l and 0.35 kU/l respectively (Phadia AB, Uppsala; Sweden).

### Sputum induction and processing

After premedication with 400 μg inhaled salbutamol administered by MDI (+ Spacer), sputum was induced by inhalation of hypertonic saline (NaCl 5%) when FEV1 post salbutamol was ≥65% predicted and isotonic saline (NaCl 0.9%) when FEV1 was <65% predicted. Saline was combined with additional salbutamol delivered by an ultrasonic nebuliser (Ultra-Neb 2000, Devilbiss) with an output set at 0.9ml/min as previously described [22]. Each subject inhaled the aerosol for three consecutive periods of 5 min and for a total time of 15 min. For safety reasons, FEV1 was monitored every 5 min and the induction stopped when FEV1 fell by more than 20% from post-bronchodilatation values.

The whole sputum was collected in a plastic container, weighted and homogenized by adding three volumes of phosphate-buffered saline (PBS), vortexed for 30 sec and centrifuged at 800 *g* for 10 min at 4°C. Supernatant was separated from cell pellet. We added DTT (dithiotreitol) to the cells which were agitated for 20 min. Cells were washed once more with PBS and resuspended in 1ml. Squamous cells, total cell counts and cell viability checked by trypan blue exclusion were performed with a manual haemocytometer. When squamous cells were >80% the sample was considered inappropriate. 90% of the samples used for our study had squamous cell count ranging from 0 to 50% [23]. The differential cell count was performed on cytospins stained with Diff-Quick after counting 400 cells.

### Sputum IgE and cytokines measurement

All induced sputum samples were concentrated by use of centrifugal evaporator. 1 ml of induced sputum was entirely airdried in a SpeedVac SC 100 centrifuge (Savant, Thermo Scientific). Afterwards the pellet was resuspended in 100 µl distilled water and mixed. Total sputum IgE was measured with ImmunoCAP system with a detection limit of 0.1 kU/l (Phadia AB, Uppsala; Sweden).

All samples were assayed for IL-4, IL-6, IL-10, IL-5, IL-17, IL-13, IFN-γ and TNF-α with the Luminex xMAP Technology by using commercially available Fluorokine MAP Kits (R&D Systems Europe Ltd, Abingdon, United Kingdom) following to the manufactureŕs guidelines and measured on a Bio-Plex 200 Platform (Bio-Rad Laboratories S.A.-N.V, Nazareth Eke, Belgium). The detection limits were 3pg/ml for IL-17, 1.5pg/ml for IL-5, 4 pg/ml for IFN-γ, 4 pg/ml for TNF-α, 2 pg/ml for IL-6, 1 pg/ml for IL-4, 11 pg/ml for IL-13 and 0.5 pg/ml for IL-10. Spiking experiments of cytokines in sputum supernatants showed that recovery ranged from 71% for IL-4 to 133% for interferon-gamma.

### Statistical analysis

Results were expressed as median (range) unless otherwise stated. Comparisons between the four groups were performed by Kruskall-Wallis Test (non parametric ANOVA) followed, in case of significance, by Dunn's multiple comparisons Test. Correlations were performed by calculating the Spearman coefficient. A P value <0.05 was considered as statistically significant.

## Results

### Patient characteristics

Demographic, lung function, airway inflammation and treatment characteristics according to sputum cellular profile are given in table 1. Exhaled nitric oxide (FeNO_50_) was higher in eosinophilic asthmatics compared to healthy subjects (p<0.05) and pauci-granulocytic asthmatics (p<0.001). FEV1 values were clearly altered in eosinophilic (p<0.01), neutrophilic (p<0.05) and pauci-granulocytic (p<0.001) when compared to healthy subjects. FVC was diminished in the neutrophilic group when compared to healthy subjects (p<0.001) and the ratio FEV1/FVC was also significantly decreased in both eosinophilic and neutrophilic asthmatics (p<0.01 and p<0.05 respectively). ACQ score was quite similar between the asthmatic groups.

The majority of our asthmatics were taking inhaled corticosteroids combined for most of them with long-acting β-2 agonists. Some of them were also receiving theophylline or leucotriene receptor antagonists (Table 1).

### Sputum IgE and cytokine levels in the all group of asthmatics

Total IgE was detectable in the sputum supernatant from the majority of subjects (70%). Overall, asthmatics had greater sputum IgE levels when compared to healthy subjects {0.3 (0–31) *vs* 0.1 (0–5.4)} (p<0.001). By contrast, there was no significant difference between asthmatics and healthy subjects regarding cytokine levels even if there was a trend for higher IL-5 levels in asthmatics {0 (0–125) *vs* 0 (0–27)} (p = 0.07).

### Sputum and serum IgE and sputum cytokine levels according to sputum cellular phenotype

**Figure 1:**
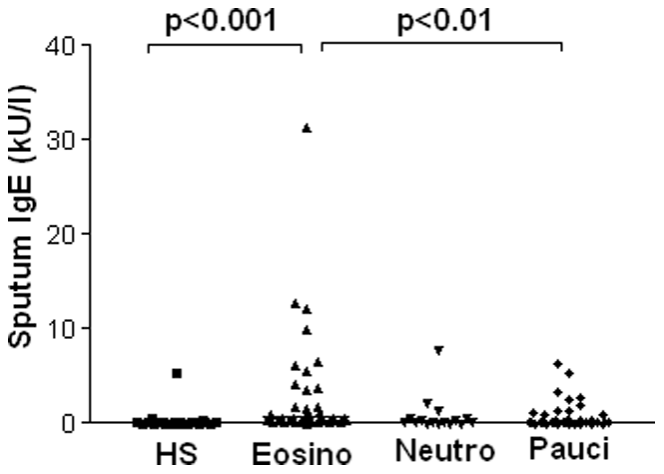
Sputum total IgE in asthmatics according to sputum cellular profile.

**Figure 2:**
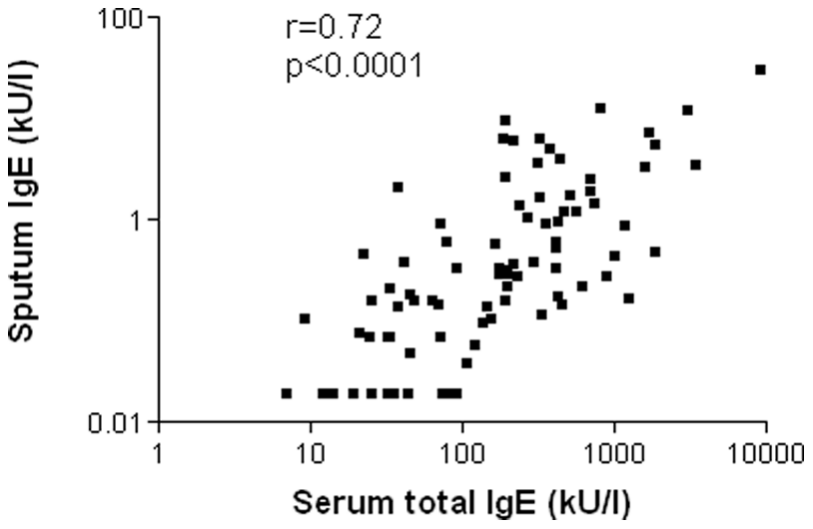
Correlation between sputum total IgE and serum total IgE in asthmatics.

When patients were classified according to their sputum cellularity, there were 41 eosinophilic (≥3%), 16 neutrophilic (>76%), 43 pauci-granulocytic and 3 mixed granulocytic. Sputum tIgE were increased in eosinophilic asthmatics when compared to healthy subjects (p<0.001) and pauci-granulocytic asthmatics (p<0.01) (Fig. 1) (Table 2). Overall sputum IgE was correlated with serum IgE (Fig 2). Serum total IgE (tIgE) were lower in neutrophilic than in eosinophilic asthmatics and healthy subjects (p<0.05 for both). Specific IgE towards classical aeroallergens or SA enterotoxins were not associated with any particular cellular phenotype (Table 2).

**Table 2:** Total sputum and serum IgE, serum specific IgE and sputum cytokine levels according to sputum cellular phenotype.

	Healthy subjects (N = 22)	Eosinophilic (N = 41)	Neutrophilic(N = 16)	Pauci-granulocytic(N = 43)
Sputum IgE (kU/l)	0.1 (0–5.4)	0.6 (0.02–31)***	0.2 (0.02–8)	0.2 (0.02–6) ††
Serum IgE (kU/l)	72 (5–195)	222 (9–9235)***	44 (7–1670)†*	125 (7–2177) *
Serum spec IgE against Staph aureus enterotoxins (kU/l)	ND	0.28 (0–23)	0.05 (0–44)	0.35 (0–2)
Serum spec IgE against Staph aureus enterotoxins (positive-%)	ND	14 (**82%**) (N = 17)	4 (**50%**) (N = 8)	11 (**79%**) (N = 14)
House dust mite (positive-%)	-	19 (**46%**)	4 (**25%**)	21 (**49%**)
Cat (positive-%)	-	10 (**24%**)	5 (**31%**)	16 (**37%**)
Dog (positive-%)	-	6 (**15%**)	5 (**31%**)	11 (**26%**)
Moulds (positive-%)	-	5 (**12%**)	3 (**19%**)	6 (**14%**)
Grass pollen (positive-%)	-	11 (**27%**)	5 (**31%**)	16 (**37%**)
Birch pollen (positive-%)	-	12 (**29%**)	2 (**13%**)	8 (**19%**)
IL-17 (pg/ml)	0 (0–11)	0 (0–51)	0 (0–99)	0 (0–17)
IL-5 (pg/ml)	0 (0–27)	6 (0–125)***	0 (0–15) ††	0 (0–40) †††
IFN-γ (pg/ml)	0 (0–0)	0 (0–13)	0 (0–0)	0 (0–192)
TNF-α (pg/ml)	8 (0–146)	5 (0–54)	7 (0–830)	4 (0–194)
IL-6 (pg/ml)	70 (12–158)	59 (0–487)	35 (2–1183)	83 (5–1002)
IL-4 (pg/ml)	0 (0–0)	0 (0–19)	0 (0–0)	0 (0–15)
IL-13 (pg/ml)	0 (0–18)	11 (0–189)*	0 (0–26)	0 (0–75) †
IL-10 (pg/ml)	ND	0 (0–3)	0 (0–0)	0 (0–21)

*p<0.05, **p<0.01, *** p<0.001 vs healthy subjects, † p<0.05, †† p<0.01, ††† p<0.001 vs eosinophilic asthmatics, ND =  not done, spec = specific. Results are expressed as median (range) except as otherwise stated.

**Figure 3:**
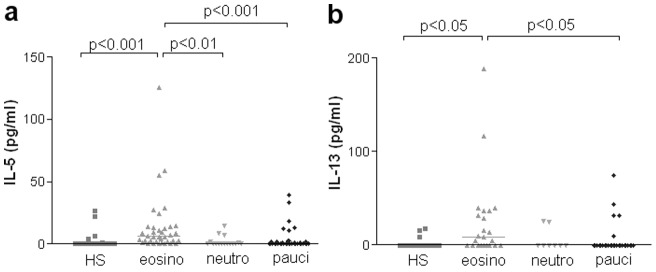
IL-5 and IL-13 levels in asthmatics according sputum cellular profile.

As far as cytokines are concerned, IL-5 was increased in eosinophilic asthmatics when compared to healthy subjects (p<0.001), neutrophilic (p<0.01) and pauci-granulocytic (p<0.001). Eosinophilic asthmatics were also characterized by greater IL-13 levels when compared with healthy subjects and pauci-granulocytic patients (p<0.05 for both) (Fig 3). No difference was found regarding other tested cytokines (Table 2).

### Patient's characteristics and cytokine levels in “IgE high” vs “IgE low” asthmatics

When asthmatic patients were classified according to their sputum IgE profile, there was no statistical difference regarding age, FeNO, spirometric values or ACQ. However, asthmatics with the “IgE high” phenotype were more atopic (p<0.01) than “IgE low” asthmatics. They were also characterized by raised sputum (p<0.001) and blood (p<0.01) eosinophils and raised blood neutrophils (p<0.05) (Table 3).

**Table 3:** Demographic, functional, airway inflammatory and treatment characteristics in “IgE high” vs “IgE low” asthmatics.

	“IgE high” asthmatics (N = 74)	“IgE low” asthmatics (N = 26)
Age (years)	47±16	48±17
Sex (m/f)	40/34	9/17
Tobacco status (ns/es/cs)	37/22/15	16/7/3
BMI	26±5	26±5
Atopy	51 (69%) **	7 (27%)
FENO_50_ (ppb)	28 (4–222)	18 (8–104)
FEV1 (%)	82±24	83±25
FVC (%)	93±19	91±21
FEV1/FVC (%)	72±13	71±12
Reversibility (%)	12±17	15±16
PC20M (mg/ml)	0.65 (0.4–14)	0.4 (0.1–14)
ACQ	1.71 (0–5.14)	1.71 (0–4.2)
Blood eosinophils (%)	4 (0.3–24) **	1.8 (0.2–7)
Blood neutrophils (%)	53 (40–72) *	63 (45–79)
Sputum eosinophils (%)	3 (0–89) ***	0.1 (0–24)
Sputum neutrophils (%)	49 (3–100)	54 (0–97)
ICS	51 (69%)	14 (52%)
ICS (eq bud/day)	1600 (400–4800)	2000 (400–4800)
LABA	41 (55%)	10 (38%)
LTRA	13 (18%)	5 (19%)
Theophylline	5 (7%)	4 (15%)
Hospi/patient/year	0.22±0.42	0.15±0.37
Exacerbation/patient/year	1.02±2.07*	0.46±0.98
Oral CS≥50% time	3 (4%)	1 (3.8%)

* p<0.05, ** p<0.01, ***p<0.001 vs IgE low asthmatics. Age, BMI, lung function, hospi/patient/year and exacerbation/patient/year are expressed as mean ± SD, PC20M as geometric mean and other parameters as median (range).

**Figure 4:**
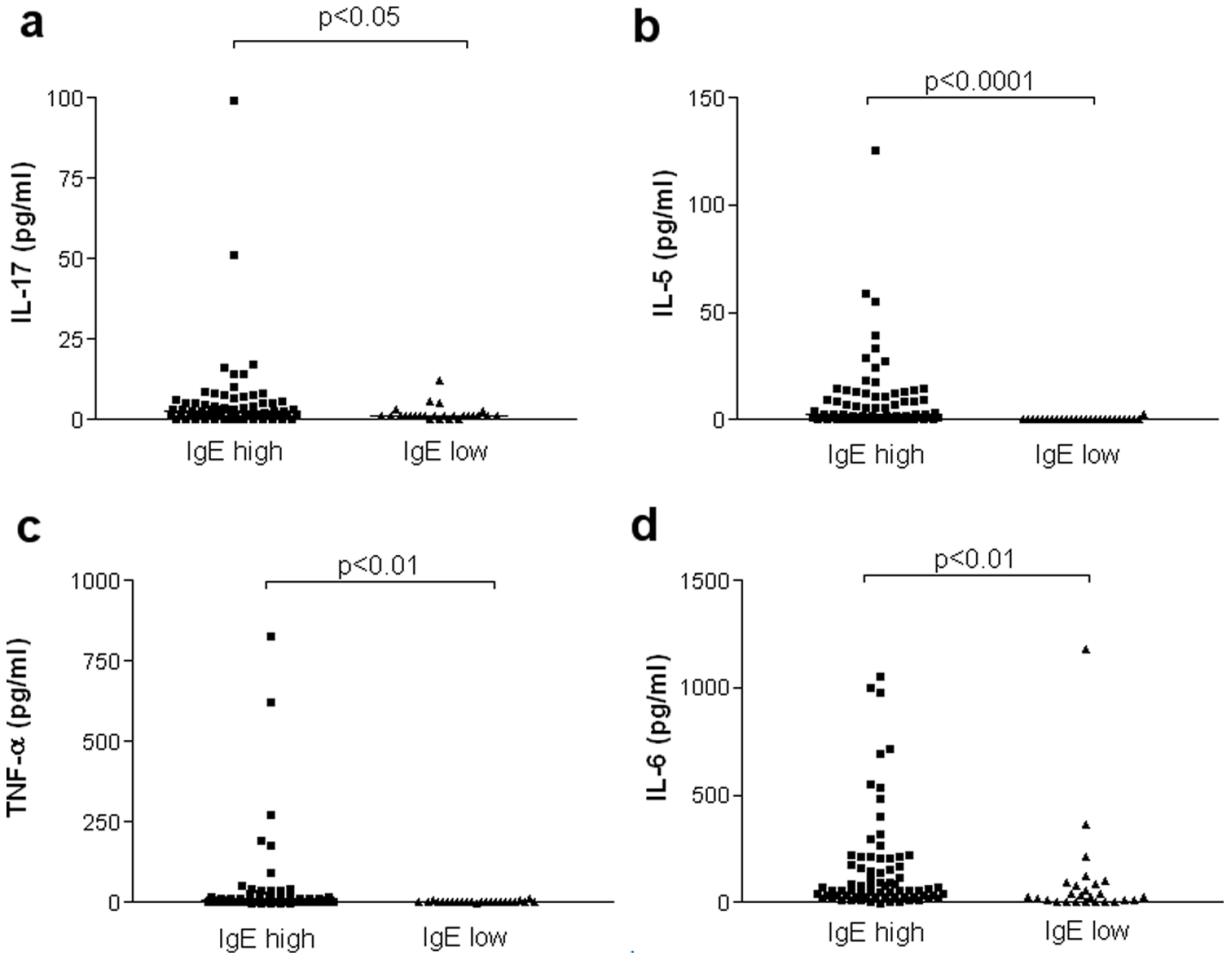
Cytokine levels in “IgE high” and “IgE low” asthmatics.

Regarding the cytokine profile, “IgE high” distinguished from “IgE low” asthmatics by raised IL-5 (p<0.0001), IL-6 (p<0.01), IL-17 (p<0.05) and TNF-α (p<0.01) from their sputum supernatant (Table 4) (Fig 4).

**Table 4:** Total sputum and serum IgE and sputum cytokine levels in “IgE high” vs “IgE low” asthmatics.

	“IgE high” asthmatics (N = 74)	“IgE low” asthmatics (N = 26)
Sputum IgE (kU/l)	0.5 (0.1–31) ****	0 (0–0)
Serum IgE (kU/l)	290 (9–9235) ****	33 (7–248)
Serum spec IgE against staph aureus enterotoxins (kU/l)	0.5 (0–44) **	0.1 (0–0.15)
Serum spec IgE against staph aureus enterotoxins (positive-%)	26 **(87%)** (N = 30)	5 **(62%)** (N = 8)
House dust mite (positive-%)	37 **(50%)**	8 **(31%)**
Cat (positive-%)	30 **(40%)** *	3 **(11%)**
Dog (positive-%)	21 **(28%)**	2 **(7%)**
Moulds (positive-%)	13 **(18%)**	1 **(4%)**
Grass pollen (positive-%)	29 **(39%)** *	3 **(11%)**
Birch pollen (positive-%)	19 **(26%)**	2 **(7%)**
IL-17 (pg/ml)	3 (0–99) *	0 (0–12)
IL-5 (pg/ml)	3 (0–125) ****	0 (0–3)
IFN-γ (pg/ml)	0 (0–193)	0 (0–0)
TNF-α (pg/ml)	6 (0–830) **	0 (0–11)
IL-6 (pg/ml)	62 (0–1051) **	27 (2–1182)
IL-4 (pg/ml)	0 (0–19)	0 (0–3)
IL-13 (pg/ml)	0 (0–188)	0 (0–37)
IL-10 (pg/ml)	0 (0–21)	0 (0–2)

* p<0.05, ** p<0.01, **** p<0.0001 vs IgE low asthmatics. ND =  not done, spec = specific. Results are expressed as median (range) except as otherwise stated.

As for serum specific IgE in these two groups of asthmatics, we found that the “IgE high” phenotype was characterized by raised serum specific IgE directed against staphylococcus aureus (p<0.01). The “IgE high” group had also more often detectable IgE towards cat (p<0.05) and grass pollen (p<0.01) (Table 4).

### Sputum and serum IgE and sputum cytokine levels according to disease severity, atopy and smoking status

Mild-to-moderate treated and untreated asthmatics as well as refractory asthmatics had higher tIgE than healthy subjects (p<0.05 and p<0.01 for refractory) but groups of asthmatics did not differ from each other. Similar to what was seen in sputum, total serum IgE were not different between the asthmatic groups. The demographic and functional characteristics of refractory asthmatics are given in Table 5.

**Table 5:** Demographic, functional and airway inflammatory characteristics according to disease severity.

	Healthy subjects (N = 22)	Mild-to-moderate untreated (N = 39)	Mild-to-moderate treated (N = 47)	Refractory asthmatics (N = 35)
Age (years)	42±13	46±16	48±17	48±12
Sex (m/f)	14/8	21/18	22/25	16/19
Tobacco status (ns/es/cs)	13/3/6	26/9/4	23/16/8	15/12/8
BMI	25±6	27±5	25±5	27±5
Atopy	0	27	26	17
FENO_50_ (ppb)	21 (6–48)	42 (8–222)*	21 (4–222)	23 (10–141)
FEV1 (%)	103±16	96±13	87±20*	62±24***†††‡‡‡
FVC (%)	108±13	103±13	96±15	80±23***†††‡‡
FEV1/FVC (%)	81±7	78±7	73±12	62±13***†††‡‡
Reversibility (%)	-	9±9	8±5	16±20
PC20M (mg/ml)	>16 mg/ml	3.02 (0.44–14.24)	2.29 (0.13–14)	ND
ACQ	ND	1.1 (0–3)	1.2 (0–4.2)	3.2 (0.9–5.2)††† ‡‡‡
Blood eosinophils (%)	1.7 (0.7–6.3)	3.5 (0.2–9)	2.9 (0.3–12.3)	3.2 (0.4–24)
Blood neutrophils (%)	53 (47–69)	52 (43–71)	53 (40–72)	60 (42–85)
Sputum eosinophils (%)	0 (0–11)	2 (0–7)**	2 (0–67)**	3 (0–89)***
Sputum neutrophils (%)	35 (0–88)	50 (3–220)	47 (5–99)	52 (0–100)

Age, BMI and lung function are expressed as mean ± SD, PC20M as geometric mean and other paramaters as median (range) * p<0.05, ** p<0.01, *** p<0.001 vs healthy subjects; † p<0.05, †† p<0.01, ††† p<0.001 vs mild-to-moderate untreated; ‡ p<0.05, ‡‡ p<0.01, ‡‡‡ p<0.001 vs mild-to-moderate treated. ND = not defined.

Atopic asthmatics distinguished from healthy subjects and non-atopic asthmatics by raised sputum IgE levels {0.36 Ku/l (0–31.2) vs 0.1 Ku/L (0–5.4) (p<0.001) and vs 0.16Ku/L (0–12.1) (p<0.05) respectively}. However a few non atopic asthmatics exhibited high sputum IgE levels. No difference was observed regarding sputum cytokine levels between atopic asthmatics and non atopic asthmatics and healthy subjects (data not shown). However, when we split “IgE high” asthmatics into atopic and non-atopic, we found that non-atopic were characterized by raised IL-5 {6 (0–125) vs 2 (0–59)(p<0.05)} and TNF-α {14 (0–829) vs 5 (0–274) (p<0.05) } and a trend for raised IL-6 (p = 0.08) when compared to IgE high atopic asthmatics. Excluding the current smokers from the asthmatic group does not alter the main findings regarding IgE and cytokines (data not shown).

## Discussion

Our study shows that eosinophilic asthmatics have higher total IgE concentrations in the sputum as compared to pauci-granulocytic asthmatics and healthy subjects. Likewise eosinophilic asthmatics exhibited a peculiar cytokine profile featuring raised Th2 IL-5 and IL-13 levels. We provide evidence for an airway “IgE high” phenotype that was associated with raised IL-5, IL-6, IL-17 and TNF-α.

There are limited data in the literature on soluble IgE in the airways of asthmatics. Our data are in keeping with the recent finding of Mouthuy et al and extend our knowledge in the field by showing that sputum IgE levels are not related to disease severity but clearly increased in those exhibiting airway eosinophilic inflammation. The role of IgE has been traditionally assigned to allergic reaction towards an aeroallergen in sensitized patients. In the nineties, Humbert et al have drawn attention to the potential role of IgE in non-atopic asthma by showing increased expression of the receptor FcεRI in the bronchial mucosa in asthmatics irrespective of the atopic status [24]. Mast cells are major effector cells in IgE dependent immediate hypersensitivity reactions and in IgE associated immune responses against certain parasites [25]; [26]. The liaison of an allergen to IgE bound at the mast cell surface is a powerful event leading to mast cell degranulation [11]. However, it is now admitted that the binding of IgE itself to its high affinity receptor at cell surface is an event sufficient to trigger cell activation [12]. More than 10 years ago, Nahm et al validated the induced sputum model as a non-invasive method for studying allergen-specific IgE antibodies in airway secretion from asthmatic patients [27]. They found that house dust mite specific IgE were detected in induced sputum supernatant from 7 of 10 house dust mite sensitive asthmatics based on skin prick tests. Moreover, a very recent study has shown that IgE production occurs both in atopic and in intrinsic asthma and that part of this IgE recognizes Der p antigens [8]. In contrast to what Mouthuy et al reported, we found that sputum IgE levels were higher in atopic than in non atopic asthmatics and that, overall, non atopic asthmatics did not distinguish from non atopic healthy subjects [8]. This, however, does not preclude the possibility that non atopic asthmatics still have greater levels of sputum IgE directed towards common aeroallergens as shown by Mouthuy et al with respect to IgE against house dust mites. Moreover we found a convincing relationship between sputum and serum IgE in our group of asthmatics suggesting that part of the sputum IgE may be related to plasma exsudation. Alternatively this might reflect a global predisposition to produce IgE in several compartments of the body.

Here, in a large group of asthmatics, we have assessed whether sputum IgE and cytokines may be related to the sputum cellular profile. Eosinophilic asthmatics clearly distinguished from healthy subjects and pauci-granulocytic asthmatics by raised sputum IgE. Our study reveals, but not explores, the mechanisms underlying this strong relationship. It is well known from bronchial allergenic challenge experiments that mast cell activation by an allergen exposure is a powerful event to stimulate eosinophil tissular recruitment [28]. We currently lack predictive factors of a good response to anti-IgE [29]. Our study supports suggestions that treatment with anti-IgE may be particularly efficient in eosinophilic asthmatics. By contrast to what is seen in eosinophilic asthmatics, neutrophilic asthmatics were characterised by lower IgE both at the airway and at the systemic level. This is in agreement with the view that neutrophilic asthma is less dependent on IgE mediated reaction but rather related to pollutant exposure or infections [30]. Here, neutrophilic asthma was defined by at least 76% neutrophils in the sputum. This was based on our own lab references values. In the literature, the definition can vary considerably according to the authors. However, performing the analysis with a threshold set at 61% for the sputum neutrophil count did not change the main message of this paper (data not shown). The reasons why there is such a variation in sputum neutrophils in healthy subjects remain controversial but may be linked to age of the population as well as its current exposure to aero-pollutants.

Regarding the cytokine profile, eosinophilic asthmatics display raised IL-5 and IL-13 sputum supernatant levels when compared with healthy subjects, neutrophilic and pauci-granulocytic asthmatics. IL-5 is a Th2 cytokine known to be able to promote eosinophil differentiation and release from the bone marrow into the blood stream. Moreover, this cytokine has also a chemotactic effect on eosinophils and enhances secretion, cytotoxicity and survival [31]. Therefore, it is not surprising that IL-5 appears to be strikingly linked to the eosinophilic pathway [32]. IL-13 is another Th2 cytokine thought to be a central mediator of inflammation in asthma. It has pleiotropic effects that mimics key features of asthma like increased smooth muscle contractility [33] or mucus secretion [34] and shares the same heterodimer receptor as IL-4 by binding to the α chain [35]. Berry M et al have investigated whether IL-13 expression and production was increased in asthma. They found greater IL-13 protein expression in bronchial biopsies by immunohistochemistry with eosinophils being the major source of IL-13 within the bronchial mucosa. Furthermore levels of IL-13 measured by ELISA were also raised in asthmatics [36]. Those findings are in keeping with our demonstration that raised sputum IL-13 levels were only found in eosinophilic asthmatics. However the fact that IL-13, like IL-5, is not increased in non eosinophilic asthma indicates that these Th2 cytokines are essentially related to a peculiar inflammatory profile rather than to asthma itself. This is in keeping with the study of Erin et al who showed that IL-5 and IL-13 were elevated in patients with severe eosinophilic asthma although in contrast to what found Erin et al we did not find an increase of IL-4 in our study [37]. Nevertheless eosinophilic bronchitis, while showing high IL-5 production, fails to discriminate from healthy subjects by increased IL-13 production [36]; [38].

It is common belief that a Th2 microenvironment is crucial in underlying atopy, this inherited predisposition to mount an IgE response towards common aeroallergens. Our data show, however, that Th2 profile is rather associated with eosinophilic inflammation than with atopy. Moreover, our data show IgE high non-atopic asthmatics were characterized by a cytokine profile featuring raised IL-5, IL-6 and TNF-α. On the other hand, it is well recognised that eosinophilic inflammation may develop in asthma irrespective of the atopic status [39]. However, it is noteworthy to highlight that the non-eosinophilic phenotype including pauci-granulocytic and neutrophilic asthma represents a major part of asthmatic population which is in agreement with a recent study by Mc Grawth et al. Our data clearly indicate that non-eosinophilic asthma is characterized by different molecular mechanisms than eosinophilic asthma. This is likely to have important consequences in terms of treatment efficacy [3]; [40].

Here we propose a new biochemical asthma phenotype based on the detection of IgE in the sputum. When patients were classified according to their IgE phenotype, we found raised IL-5, IL-6, IL-17 and TNF-α in the “IgE high” asthmatic subgroup. The role of IL-5 in asthma and its relationship with eosinophils has already been discussed (see above). Besides, IL-5 together with IL-6 can promote IgE synthesis and increases IL-4-dependent IgE synthesis [41]. It may appear somewhat surprising that IL-17 was associated with the high sputum IgE and not with the neutrophilic phenotype as IL-17 has been shown to promote neutrophil recruitment and activation [42] and as some patients with hyper-IgE syndrome were shown to be deficient in IL-17 secreting T cells as a result of STAT3 mutation [43]. Our finding also contrast to what Bullens et al reported using sputum mRNA but mRNA and proteins levels are not necessarily tightly related [44]. The reason why IL-17 was not associated with neutrophilic inflammation in our study is not clear but our data point out the fact that Th2 and Th17 pathways may be operating together in those asthmatics exhibiting high IgE levels in their airways. The association between the high IgE phenotype and high TNF-α and IL-6 sputum levels is in keeping with the fact that mast cells are a potent source of TNF-α and IL-6 that may be activated by monomeric IgE [12]. Treatment with anti-TNF-α has generally proved rather disappointing in asthma [45]; [46]although some reports had shown convincing and promising responses [47]; [48]. Our study shows that those patients with high IgE in the sputum might perhaps be suitable targets for anti TNF-α treatment. In our hands, cytokines like IL-4, IFN-γ and IL-10 were undetectable in the majority of patients. Sputum processing with a mucolytic agent may influence the level of cytokines measured in the supernatant [49]. In our study we cannot, however, incriminate the use of DTT as the supernatant was only diluted with PBS, the mucolytic agent being reserved to the cellular part for improving the quality of cytospins. Furthermore, recovery from spiking experiments was excellent indicating that the poor detection of some of them cannot be accounted for by cytokine trapping in sputum supernatant heterogeneous milieu.

## Conclusion

Our study shows that asthmatics have raised sputum IgE levels associated with the eosinophilic phenotype and that the airway “IgE high” is characterized by a global cytokine overproduction not limited to a Th2 profile. Overall, our study point to the cellular and molecular heterogeneity in asthma, which calls upon targeted treatments. If new biologicals for asthma treatment have to fulfil our expectations, they certainly must be tested in selected asthmatic population.
